# Metabolomic Analysis of Wooden Breast Myopathy Shows a Disturbed Lipid Metabolism

**DOI:** 10.3390/metabo13010020

**Published:** 2022-12-22

**Authors:** Gavin M. Boerboom, Alberto Navarro-Villa, Theo A. T. G. van Kempen

**Affiliations:** 1 Trouw Nutrition R&D, 3811 MH Amersfoort, The Netherlands; 2 Department of Animal Science, North Carolina State University, Raleigh, NC 27695, USA

**Keywords:** broiler, metabolomics, myopathy, wooden breast

## Abstract

Myopathies have risen strongly in recent years, likely linked to selection for appetite. For white striping (WS), causes have been identified; but for wooden breast (WB), the cause remains speculative. We used metabolomics to study the breast muscle of 51 birds that were scored for both at 35 days of age to better understand potential causes. A partial least square discriminant analysis revealed that WS and WB had distinct metabolic profiles, implying different etiologies. Arginine and proline metabolism were affected in both, although differently: WB increased arginine in breast muscle implying that the birds did not use this pathway to increase tissue blood flow. Antioxidant defenses were impeded as shown by low anserine and beta-alanine. In contrast, GSH and selenium concentrations were increased. Serine, linked to anti-inflammatory properties, was increased. Taurine, which can stabilize the cell’s sarcolemma as well as modulate potassium channels and cellular calcium homeostasis, was also increased. Mineral data and depressed phosphatidylethanolamine, cAMP, and creatine-phosphate suggested compromised energy metabolism. WB also had drastically lower diet-derived lipids, suggesting compromised lipid digestion. In conclusion, WB may be caused by impaired lipid digestion triggered by a very high appetite: the ensuing deficiencies may well impair blood flow into muscle resulting in irreparable damage.

## 1. Introduction

Poultry meat is highly regarded as one of the most nutritious protein sources. The demand for poultry meat has risen in recent decades due to the low cost, good nutritional profile (high-protein content coupled with a balanced n-6 to n-3 polyunsaturated fatty acids ratio, low fat, low cholesterol, and presence of some functional components), suitability for further processing and no cultural or religious restrictions [1,2,3]. Additionally, together with milk, poultry products are recognized as one of the most environmentally efficient livestock products with regard to carbon footprint and resource depletion [4]. As for any product, the environmental impact is strongly influenced by the total amount and quality of the finished food product [5]. Thus, the growing demand for chicken meat and the push for lower production costs has led to the genetic selection of broilers for quantitative traits. It is worth noting that the selection procedures in favor of fast-growing broiler chicken strains have led to an extraordinary improvement in animal performance [6]. This significant increase in growth rate has been achieved by inducing muscle hypertrophy; and, unintentionally, genetic selection might have also induced meat quality issues such as white striping (WS) and wooden breast (WB) [7,8,9]. The alterations in the chemical composition and technological properties of the meat from birds affected by these myopathies may prevent the final product from reaching the consumer, and, therefore, increase wastage, thereby compromising sustainability [10,11,12]. The incidence of these myopathies has been estimated to lead to an economic loss of more than USD 200 million/year in the United States [13]. White striping is recognized by white striations that run parallel to the muscle fiber [14]. The underlying etiology of WS has been previously described by our group, and the proposed mechanism has been echoed by others [9,15,16].

Unfortunately, information around the underlying etiology of WB is less clear in the literature. WB is an abnormality in which the *Pectoralis major* muscle in broilers presents a pale and hardened consistency in cranial and/or caudal areas often accompanied by petechial hemorrhages and exudate [17,18]. Affected muscle have moderate to severe multifocal regenerative myodegeneration and necrosis with a variable amount of interstitial connective tissue accumulation or fibrosis. WB incidence has increased over the last years and, unfortunately, fillets with this abnormality are rejected from human consumption completely. The lack of automated systems at the slaughterhouse to detect WB complicates the quantification of this condition. However, a recent study using near infrared spectroscopy reported a prevalence of 9% among 10,483 fillets from high-yielding strains targeted to reach body weights of 2.72–4.53 kg [19]. WB fillets also show pathological changes including a reduction in protein and ash as well as an increase in fat and collagen [20]. It is often assumed that a link exists between the etiology of WS and WB, as both are frequently present together [13,21]. It is generally accepted that the prevalence of WB correlates positively with feed intakes and thus growth rates at a given age [22]. Studies have seen incidences in WB at 9 weeks of age of 85%, with more than 42% ranking severe or very severe; but already at 3 weeks of age, alterations in lipid-related genes are observed [23]. WB has been linked to rapid muscle growth inducing oxidative stress or hypoxia due to insufficient vascularization [3]. Since there are still many uncertainties about the underlying etiology, metabolomics was used to analyze both unaffected as well as affected breast muscle tissue. The overall objective of this study was to use non-targeted metabolomics to identify biological pathways that are involved in the underlying etiology of WB in broiler chickens.

## 2. Materials and Methods

### 2.1. Animals and Sample Collection

Sample collection and analysis has already been described earlier [15]. In brief, broilers used in this study were all ROSS 308 males, raised on a research farm (Poultry Research Centre; Casarrubios del Monte, Toledo, Spain) to 35 days. Males were chosen as they typically have a faster growth rate and higher incidence of myopathies. Diets fed were in line with local commercial practices (Table 1).

On day 35, birds were euthanized in compliance with welfare rules. Time between euthanization and sample collection was as short as possible to ensure proper sample quality (on average 2–2.5 min). Muscle samples of the pectoralis major muscle were collected for 51 birds, which were randomly selected (two birds per pen). These muscle samples were scored for WS and WB. For WB, filets that were flexible throughout were scored as “normal” while filets in which hardness was present were scored as “moderate/severe” based on criteria described in the literature [24]; moderate/severe will be referred to as severe below. The samples were frozen using liquid nitrogen and stored at −80 °C until further processing.

An additional 47 birds were collected from a trial using diets with a similar nutrient composition. Those birds (mixed sex, Ross 308, 42 days of age) were scored for WS and WB as outlined above, and muscle samples were analyzed for vitamin K_2_ (MK-7) using LC-MS. Samples were grinded and 20 mL of acetonitrile was added. This was mixed for 60 min and then analyzed.

### 2.2. Sample Analysis

Metabolomic analysis of the 51 samples was performed by Metabolomic Discoveries (Potsdam-Golm, Germany), which is described in more detail by Boerboom (Boerboom et al., 2018). Muscle samples were also analyzed for phosphorus (P), sodium (Na), potassium (K), calcium (Ca), magnesium (Mg), copper (Cu), iron (Fe), zinc (Zn), cobalt (Co), nickel (Ni), and selenium (Se). Samples were weighed, destructed using 16 N HNO_3_, and then analyzed by inductively coupled plasma mass spectrometry using a NexION 350 D (Perkin-Elmer, Waltham, MA, USA) according to method NEN-EN 15510 [25].

### 2.3. Data Analysis

Metabolomics data were analyzed using MetaboAnalyst (Xia Lab, McGill, Ste. Anne de Bellevue, Quebec, Canada) [26,27,28,29,30]. Data were normalized on internal standards, log transformed and auto-scaled to remove heteroscedasticity and reduce bias. The *p*-value was adjusted using the false discovery rate (FDR) correction and was deemed significant if *p* < 0.05 [31]. Pathway analysis was done using MetaboAnalyst. The significance was calculated using pathway enrichment analysis which groups functionally related metabolites and tests whether they are significantly enriched. The impact was calculated by taking the pathway structure into account, thereby assigning more impact to changes in key positions in a pathway. As a measure for this, betweenness centrality was chosen, which takes into consideration the global network structure and not only the immediate neighbor of the metabolite like degree centrality does [30,32]. Additionally, a Partial Least Squares—Discriminant Analysis (PLS-DA) was performed using JMP 16.2 Pro (SAS Inst., Cary, NC, USA) using both WB and WS as discrete response parameters. Full cross validation and two rounds of very important variable selection (threshold > 1) were used. Data were also analyzed by multi-factor ANOVA using WS, WB, and their interaction WS × WB. Vitamin K_2_ data were analyzed using ANOVA including WB, WS, gender, and their 2-way interactions; this model was simplified prior to final analysis to only WB, as other factors were not significant (*p* > 0.2).

## 3. Results

Birds with severe WB averaged 2500 ± 62 g at sampling (mean ± SEM; day 35), birds scored as normal averaged 2380 ± 28 g (*p* = 0.08). The animals not selected were weighed on a pen basis and averaged 2289 ± 20 g; daily gain averaged 64.5 ± 0.58 g/d, feed intake averaged 94.5 ± 0.76 g/d, and FCR averaged 1.466 ± 0.005.

**Figure 1:**
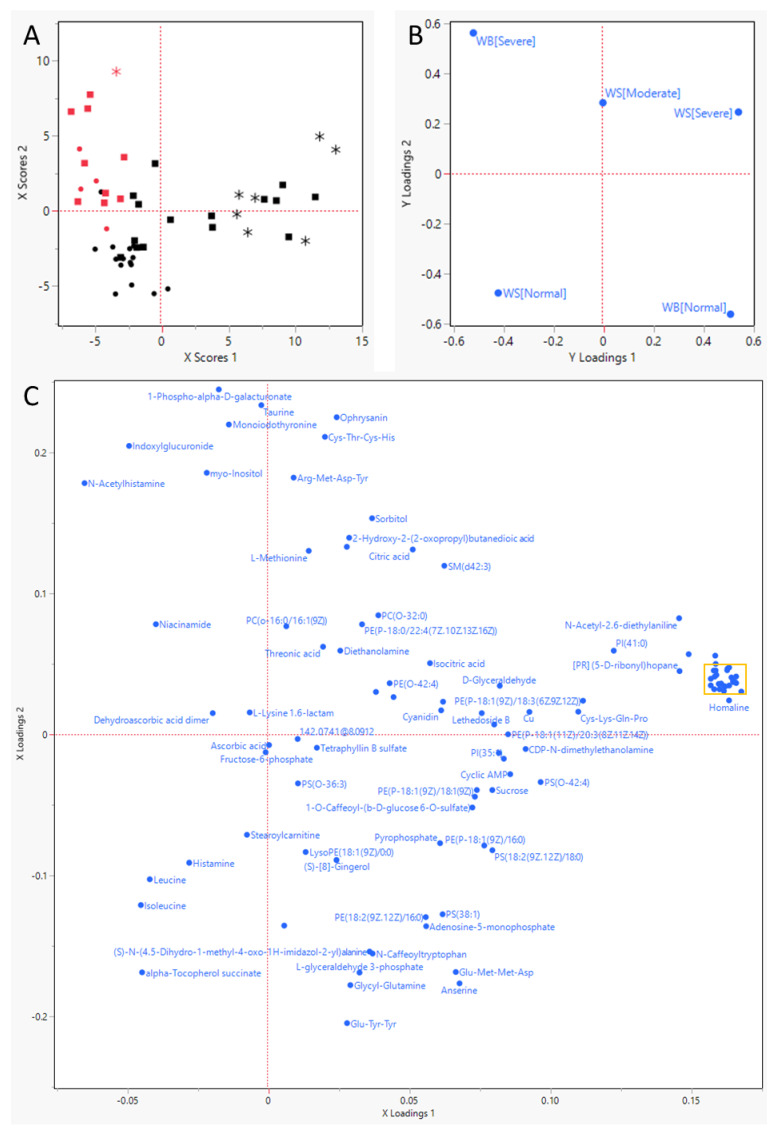

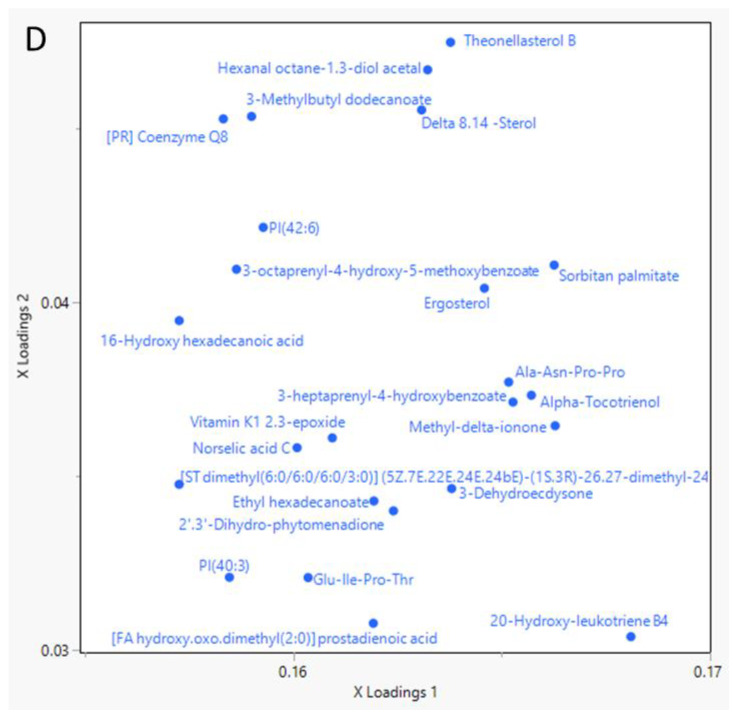
Partial-Least Squares—Discriminant Analysis results. (**A**) score plot for the individual birds, represented by red (severe WB) or black (no WB) symbols; circles (• or •) indicate that they had no WS, squares (▪ or ▪) moderate WS, and stars (* or *) severe WS. (**B**) Y Loadings plot for the response parameters, severity of Wooden Breast (WB) and White Striping (WS). This plot uses the same layout as plots (**A**,**C**) and shows how the response variables (WB and WS) associate with the individual animals (plot (**A**)) and the various metabolites that survived the very important parameter selection (plot (**C**)). (**C**) Loadings plot for the metabolites remaining after two rounds of very important parameter selection). (**D**) close-up of the yellow rectangle in (**C**).

Out of a total of 51 samples (Table 2), 14 samples (27%) received a WB score of severe. Moderate WS was observed in 24 samples (47%), and severe WS was observed in eight birds (16%). Ten birds exhibited both WB and WS (of which one severe WS). The PLS-DA analysis (Figure 1) indicated that WB was placed effectively orthogonal to WS.

In the metabolomics data, bias was removed by the pre-processing steps described before [15], limiting the difference in absolute magnitude and difference in variance, thereby improving the quality of the dataset. The total number of annotated metabolites used in the analysis was 497. An ANOVA was used to determine the significant differences between the metabolites in normal birds and WB-affected birds. Afterwards, relative fold change was taken into account, and a volcano plot was created. The cut-off was set at an FDR-corrected *p*-value of 0.05 and a fold change threshold of 2. This yielded 65 metabolites (Supplementary Table S1). A positive fold change indicates that the severe WB group had increased concentrations of metabolites compared to the normal group. Several metabolites quantified were plant metabolites with poorly defined metabolic function. These will not be discussed in detail. It is worth noting that there is a high number of small peptides and of fat-soluble components that appear to be affected by the occurrence of WB.

Pathway analysis (Table 3) showed that histidine metabolism was most significantly affected. The highest impacted pathway was the taurine and hypo-taurine metabolism, which is somewhat confounded by the method applied, as these pathways are small and as such will more easily rank high. Individual pathway visualization was performed for the impacted pathways to ensure a more precise interpretation of the data. This highlighted the metabolism of arginine and proline, taurine, β-alanine, and glutathione. The pathways with a low impact were removed due to their lower biological effect.

**Figure 2:**
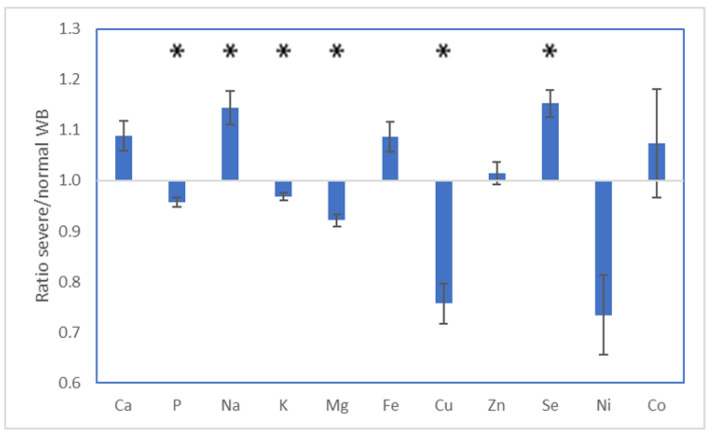
Effect of WB on tissue mineral level. Ratios are in relation to the normal WB group (* *p <* 0.05).

The results of the mineral analysis indicated significant changes occurring between normal and WB-affected breast tissues (Figure 2). The levels of sodium and selenium were elevated in WB-affected tissues (*p* < 0.05), while the levels of phosphorus, potassium, magnesium, copper, and nickel were lowered in WB-affected tissues (*p* < 0.05).

**Figure 3:**
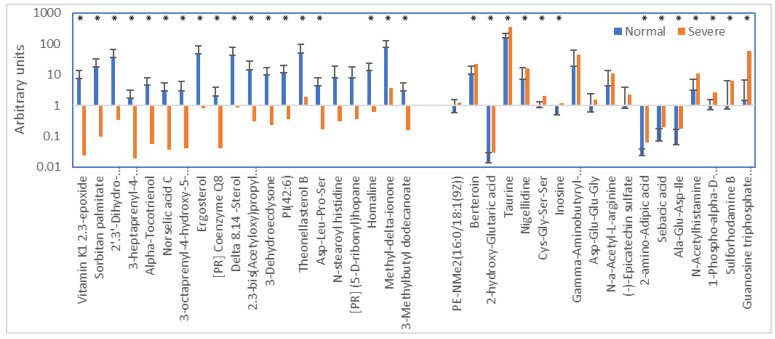
Metabolites most strongly decreased (<5% vs. normal) and most strongly increased (>100% vs. normal) as a result of WB (* *p* < 0.05).

The ANOVA analysis indicated that approximately 20 metabolites were decreased by over 95%, while 18 increased by more than 100% (Figure 3), most of them significantly so (such drastic downshifts were not seen in WS). In Figure 3, it is apparent that the compounds decreased are mainly lipophilic compounds with a uniquely dietary and/or microbial origin; several of these lipophilic compounds clustered strongly in Figure 1D. The most strongly increased metabolite was guanosine triphosphate adenosine and also taurine and the lysine catabolite 2-amino adipic acid stand out.

**Figure 4:**
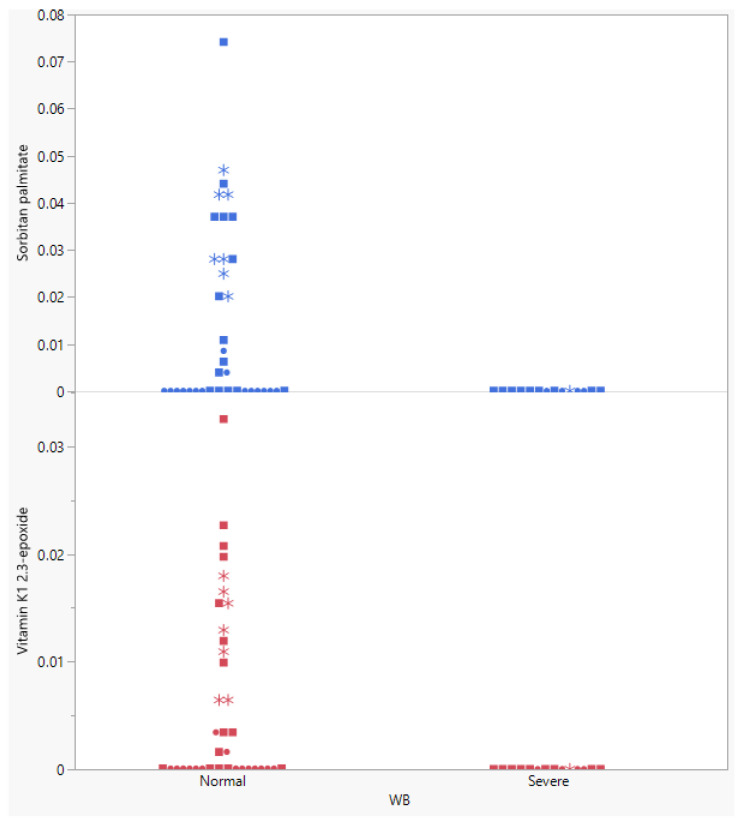
Two of the metabolites most severely depressed in birds with Wooden Breast (WB). Affected birds registered 0 for both of those metabolites, while normal birds displayed a wide spread in concentration. Circles (•) indicate that birds had no White Striping (WS), squares (**▪**) moderate WS, and stars (*) severe WS.

An in-depth look at the two metabolites with the strongest drop, vitamin K_1_ 2.3-epoxide, and sorbitan palmitate (Figure 4), shows that both metabolites were effectively 0 in all birds with WB. In the normal birds, a wide range of concentrations was found.

**Figure 5:**
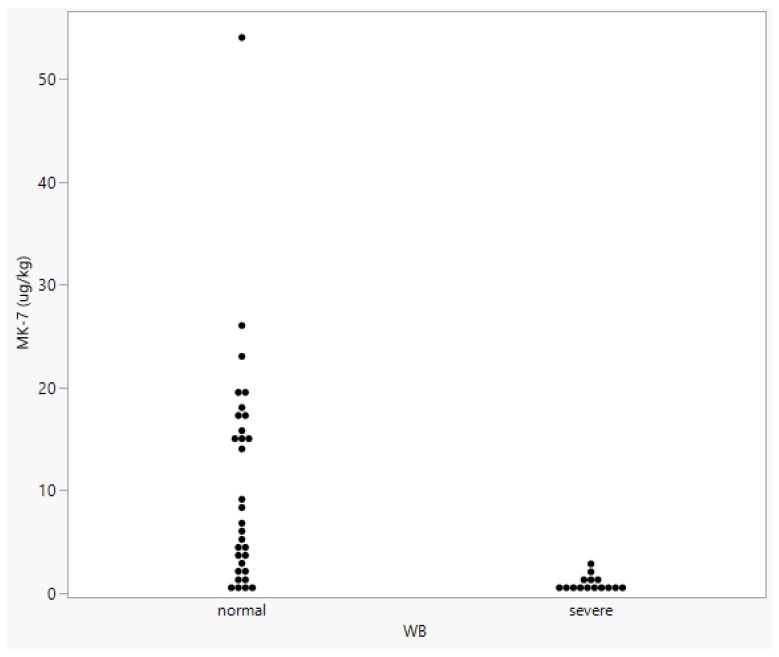
Vitamin K_2_ (as MK-7) measurement in a separate group of birds scored for Wooden Breast (WB).

To further investigate a vitamin K_2_ deficiency as a possible factor in WB, we measured vit. K_2_ in birds collected in a separate trial (47 birds, of which 15 had WB; Figure 5). Neither sex nor WS affected vit. K_2_ levels. Birds without visible WB again showed a wide spread in vit. K_2_ levels, while in the affected birds the majority did not have detectable vit. K_2_ (Control: 10.6 µg/kg, WB: 0.8 µg/kg, *p* = 0.0003).

## 4. Discussion

Meat myopathies such as WS and WB are of huge concern to the broiler industry. Their occurrence is linked to the high appetite and consequently fast growth of the current genetic strains of birds used, and the resulting compromised blood supply and hypoxia are commonly brought forward as a starting point for their development [9,33]. The results of the PLS-DA analysis in the current study imply that WB and WS do not share a similar metabolic profile, but rather, express different changes in metabolite profiles, even if pathophysiological similarities might occur between the two diseases. Our results also imply that the metabolomics profile can be used to differentiate the two groups, in line with observations in the literature [34].

Arginine and proline metabolism were shown to be affected by WB occurrence, apparently like previous findings for WS [15]. However, the metabolites that changed within these pathways for WS were not the same metabolites as the ones changed in WB. Arginine to citrulline conversion was hypothesized to be increased in WS due to the observation of lower arginine levels and higher citrulline levels in WS cases [15]. In contrast, arginine levels showed an increase in WB-affected tissues (*p* < 0.05). This appears to indicate that there was still arginine available for conversion to citrulline for production of nitric oxide, required for enhanced blood flow [35,36]. Arginine in animals is used in two direct metabolic pathways: 1. Arginine is decomposed into ornithine and urea by arginase, and 2. Arginine and molecular oxygen generate citrulline and nitric oxide (NO) by nitric oxide synthase (NOS) [37]. However, no change in ornithine or citrulline was observed, indicating that none of the previously cited metabolic pathways of arginine utilization were being used.

Anserine (beta-alanyl-methyl-L-histidine) was reduced in WB-affected tissues (*p* < 0.05). Anserine is important for antioxidant functions, pH buffering and anti-glycation agents [38,39]. Anserine levels tend to be lower when birds get older resulting in reduced antioxidant capacity [40]. This reduction is in accordance with literature on WB, where a reduction in anserine has been observed more frequently [9,41]. A potential contributor to the lower levels of anserine that were observed in this study could be the lower levels of beta-alanine, which was also observed (*p* < 0.05). The rate of anserine synthesis in skeletal muscle is dependent on the amount of circulating beta-alanine. In line with these findings, a recently published article on metabolomics in WB-affected birds indicated that 3-methyl-histidine was the top metabolite being affected in plasma, with eight more metabolites involved in histidine metabolism being affected, further indicating the effect WB occurrence has on histidine metabolism [34]. Surprisingly, though, a study feeding high levels of histidine indicated no effects on meat quality, even though a higher level of carnosine was observed [42].

Phosphatidylethanolamine (PE) is the second most abundant glycerophospholipid in eukaryotic cells [43]. It has diverse cellular functions that include serving as a precursor for the formation of phosphatidylcholine, influencing membrane topology and promoting cell and organelle membrane fusion, oxidative phosphorylation, mitochondrial biogenesis, and autophagy. It is also an important precursor for other lipids [43,44,45]. The results of the metabolomics indicate that the levels of PE in WB-affected tissues are lowered. In case of limited availability, mitochondrial activity is negatively affected given the importance of PE in mitochondrial stability and activity [43]. This could have compromised cellular energy metabolism in WB-affected tissues, in line with lowered levels of cAMP and phospho-creatine. Furthermore, it has been observed that the level of PE is important for membrane stability, and as such, the lower levels of PE observed could have led to membrane destabilization, leading to WB [46]. This is further substantiated by the mineral results, which show a reduction in phosphorus, magnesium, and copper along with an increase in sodium. These changes fit with an impairment in metabolic activity of the cell as phosphate and magnesium are key in energy metabolism while copper is an important cofactor for various oxidases. Perturbed energy metabolism can subsequently lead to an efflux of potassium and an influx of calcium (ns) and sodium into the cells [47]. In order to compensate for this, the cells might have produced high amounts of glutathione (GSH) as this is a master regulator of metabolism within the cell [48]. Aside from its function as a regulator of metabolism, it is also a cysteine-derived antioxidant and important for detoxification, protection from oxidative stress and maintenance of redox balance. In line with this antioxidant effect, a significant increase in selenium was observed in WB birds. Synthesis of GSH is driven by the activity of the pentose phosphate pathway [49], which has been shown to be influenced by WB in previously published literature [34,41]. They indicated that WB affected purine metabolism, which was a result of the increased activity of the pentose phosphate pathway or a decreased nucleotide utilization. In this dataset, serine levels tended to be higher in WB-affected tissues (*p* = 0.05) and they play an important role in the metabolism of purines and pyrimidines, further substantiating the effect WB has on purine metabolism [50]. Numerous studies have indicated that glycine can exert anti-inflammatory and antioxidant effects, protecting against hypoxia induced injuries, albeit in renal tissue [51]. It could also be that serine expression is increased in WB-affected tissue to protect against the hypoxic state that is created by damaged vascular support system [17]. The conversion of serine into glycine leads to an increase in Ca influx, something that is also observed in the WB-affected tissues in this study (Figure 2).

As described for WS, taurine is often released at higher levels to protect against damage of hypoxic nature, and in addition, it plays a pivotal role in Ca homeostasis [2,15]. Levels of taurine, a metabolite of methionine, increased similarly for both WB and WS (*p* < 0.05). The key effect of taurine is stabilization of the sarcolemma. Besides this, taurine also modulates the activity of various types of K channels and, in particular, those able to couple metabolic state of striated fibers to electrical activity. Lastly, it controls intracellular calcium homeostasis by modulating calcium handling mechanisms and consequently excitation-contraction coupling [52]. Taurine uptake into cells also has an osmotic function, and high levels may reflect swollen cells, which is a common observation in WB-affected tissues [53]. Another affected metabolite in the cysteine and methionine metabolism is 3-methylthiopropionic acid, which is almost absent in the WB-affected tissue (*p* < 0.01). 3-methylthiopropionic acid is an intermediate in the transamination pathway of methionine metabolism. As methionine was not affected, a possible explanation for low levels of 3-methylthiopropionic acid is that the transamination pathway for methionine catabolism was downregulated and methionine instead was converted to taurine, in line with increased taurine levels [54].

The drastic reduction in various diet-derived lipids as observed in the ANOVA analysis implies that lipid digestion was impaired, which may well be linked to the very high feed intake of birds affected by WB. Indeed, Nir et al. (1973) and Krogdahl (1985) showed that digestion of fat is typically first limiting when intestinal passage rate is increased [55,56]. This observation also aligns with changes in lipid-related genes [23,57]. Liu et al. also showed strong changes in lipids, but their analysis focused on lipids that are not per se obtained from the diet [58]. Several of the affected lipids are linked to specific metabolic functions, like a vitamin, in either the bird or in the feedstuffs the birds consumed. These lipids may well be harder to digest than those used as energy sources, aggravated by very high feed intake. For example, for vitamin E a bioavailability of only 5% was obtained [59]. In line with this, Thompson (1989) highlighted numerous case reports describing patients developing deficiencies of vitamins D, E, and K as a consequence of lipid malabsorption [60]. As shown in Figure 4, birds with WB had effectively tissue levels of zero for the most severely affected lipids. In the ‘normal’ birds, interestingly, also several birds registered at 0, but also a substantial number of birds had higher levels. A possible explanation, in line with field observations, is that deficiencies of these diet-derived lipids and thus problems with WB start earlier in some birds than others; those that appeared normal at slaughter may well have developed WB if they had been slaughtered a week later due to aggravating deficiencies of these lipids. A vitamin K_2_ analysis of 47 samples of breast muscle tissue obtained from an independent flock of birds confirmed that birds with severe WB had practically zero vit. K2, while normal birds again had a wide spread in vit. K_2_ levels (Figure 5). Vit. K_2_ is involved in calcium metabolism; when animals are deficient, it leads to calcium deposits in blood vessels, in line with what Sihvo and Mutryn showed, and in line with data from Abasht et al. (2021) showing vascular endothelial dysfunction [17,57,61,62]. This may well impede blood flow into breast muscle tissue, resulting in atrophy, in line with the observations that creatine kinase (a marker for muscle injury) increased with WB severity [63]. Several of the observations in the metabolic pathways fit with this observation as does the appearance of petechia and hemorrhages.

Another interesting lead is the enormous increase in guanosine triphosphate adenosine (Figure 3; also seen in WS). This class of molecules is used to cap mRNA protecting it from degradation [64]. In addition, they are strong agonists of the purinergic system involved in vasoregulation [65]. High levels may well mean that the animal was trying to counter hypoxia by vasodilation and vascular regeneration, but unsuccessfully did so as blood vessels were damaged by calcium deposits linked to a vitamin K_2_ deficiency.

## 5. Conclusions

Taken together, the results of our analyses seem to indicate that birds suffering from WB suffer from a problem with the digestion of lipids such as vitamin K_2_. Although not evaluated in this experiment, these digestive problems could potentially be the result of feed intake that exceeds the digestive capacity of the birds. The resulting lipid deficiencies may well impair vascular health and antioxidant defenses, resulting in tissue hypoxia and irreparable damage. Tissue hypoxia is something that WS and WB have in common, but the magnitude/extend of the hypoxic state as well as the expression do differ. In the case of WS, the hypoxia appears more manageable, allowing the tissue to retain some functionality and growth, albeit with an altered nutrient profile. In the case of WB, the hypoxia appears more severe resulting in tissue that has severely compromised functionality, in line with other observations [66]. Problems with fat digestion as a result of excessive feed intake deserve further attention and, hence, should be investigated further.

## Figures and Tables

**Table 1:** Ingredient and nutrient composition of the experimental diets as fed to the birds sampled for metabolomics. Data are in % except where noted.

Ingredient Supplementation (%)	Starter Phase (d0–7)	Grower Phase (d7–25)	Finisher Phase (d25–35)
Wheat	52.63	56.00	61.70
Soybean meal 47	28.77	25.89	21.51
Maize	10.00	8.81	8.00
Soy oil	4.41	4.25	4.37
Glycerin 99.5%		2.00	2.00
Soycomil		0.20	
Monocalcium phosphate	1.24	0.63	0.32
Calcium carbonate,	1.17	0.54	0.45
Sodium bicarbonate	0.28	0.22	0.24
NaCl	0.15	0.16	0.16
L-lysine∙HCl	0.30	0.27	0.29
DL-methionine	0.23	0.21	0.19
L-threonine	0.066	0.070	0.076
Xylanase (Axtra XB)	0.10	0.10	0.10
Phytase (Phyzyme XP TPT)	0.10	0.10	0.10
Maxiban 16%	0.063		
Elancoban 20%		0.050	
Premix ^1^	0.50	0.50	0.50
Calculated Nutrient values			
Crude protein	21.7	20.4	19.4
AME broilers (kcal/kg)	2850	2922	3000
dLys	11.5	10.60	9.80
dMet	5.03	4.69	4.30
Ca	9.0	5.50	4.5
dP	4.6	3.50	2.90

^1^ Provided per kilogram of complete diet: vitamin A, 7500 IU; vitamin D3, 1500 IU; vitamin E, 6 IU; vitamin K_3_, 2.0 mg; vitamin B_1_, 2.0 mg; vitamin B_2_, 3.0 mg; vitamin B_6_, 3.0 mg; vitamin B_12_, 0.03 mg; niacinamide, 20 mg; D-pantothenic acid, 6.5 mg; folic acid, 0.5 mg; biotin, 0.1 mg; choline, 295 mg; iron, 40 mg (as FeSO_4_•7H_2_O); copper, 12 mg (as CuSO_4_•5H_2_O); manganese, 90 mg (as MnSO_4_•H_2_O); zinc, 60 mg (as ZnO); iodine, 1.0 mg (as CaI); selenium, 0.20 mg (as Na_2_SeO_3_•5H_2_O); (supplied by Trouw Nutrition Spain).

**Table 2:** Bird count for the various myopathy scores.

		WS			
		Normal	Moderate	Severe	WB Total
WB	Normal	15	15	7	37
	Severe	4	9	1	14
	WS total	19	24	8	51

**Table 3:** Pathway analysis results using MetaboAnalyst. The *p*-value was calculated using pathway enrichment analysis and the impact was calculated using pathway topological analysis using betweenness centrality and using Gallus Gallus as a pathway library.

Pathway Name	*p*-Value (FDR Corrected)	Impact
Histidine metabolism	0.002106	0.54917
Starch and sucrose metabolism	0.019798	0.22073
Beta-alanine metabolism	0.019798	0.51119
Taurine and hypotaurine metabolism	0.023189	0.71428
Arginine and proline metabolism	0.026478	0.48672
Glutathione metabolism	0.038001	0.37762
Glycerophospholipid metabolism	0.042407	0.43635
Cysteine and methionine metabolism	0.042453	0.44302
D-Glutamine and D-glutamate metabolism	0.043772	0.50000

## Data Availability

The data are not publicly available due to privacy.

## Supplementary Materials

The following supporting information can be downloaded at: https://www.mdpi.com/article/10.3390/metabo13010020/s1, Table S1: Significantly affected metabolites based on volcano plot between the groups (FDR corrected *p* < 0.05, Log2FC threshold is 1).
