# Supplementary material for: Metabolomic Analysis of Wooden Breast Myopathy Shows a Disturbed Lipid Metabolism
Source: Metabolites. 2022 Dec 22;13(1):20. doi: 10.3390/metabo13010020 (PMC9862534; doi:10.3390/metabo13010020)
Supplement: Supplementary file 1 [file metabolites-13-00020-s001.zip › metabolites-2075827-supplementary.pdf]

metabolites

Supplemental Table S1. Significantly affected metabolites based on volcano plot between the groups (FDR-corrected P<0.05, Log2FC threshold is 1).

| | log2(FC) | P-value |
|---|---|---|
| 3-Dehydroecdysone | -4.7808 | P<0.001 |
| Homaline | -3.822 | P<0.001 |
| 20-Hydroxy-leukotriene B4 | -3.4207 | P<0.001 |
| Leucyl-Alanine | -1.0919 | P<0.001 |
| His-Met-Trp-Ser | -1.0455 | P<0.001 |
| Phenylalanyl-Serine | -1.1125 | P<0.001 |
| Alanyl-Tryptophan | -1.0207 | P<0.001 |
| Hexanal octane-1.3-diol acetal | -3.7221 | P<0.001 |
| FA hydroxy.oxo.dimethyl20 prostadienoic acid | -3.4568 | P<0.001 |
| Serinyl-Hydroxyproline | -1.0087 | P<0.001 |
| Sucrose | -3.1052 | P<0.001 |
| .Glu-Ile-Pro-Thr | -3.469 | P<0.01 |
| Hydroxymethylphosphonate | -1.3663 | P<0.01 |
| Ethyl hexadecanoate | -3.3231 | P<0.01 |
| Phosphocreatine | -2.9579 | P<0.01 |
| Threoninyl-Glycine | -1.0555 | P<0.01 |
| 2'.3'-Dihydro-phytomenadione | -6.5216 | P<0.01 |
| Methyl-delta-ionone | -4.2539 | P<0.01 |
| Alpha-Tocotrienol | -6.181 | P<0.01 |
| Berberrubine | -1.5769 | P<0.01 |
| Vitamin $K_1$ 2.3-epoxide | -7.835 | P<0.01 |
| Fructose | -1.8442 | P<0.01 |
| Norselic acid C | -5.8677 | P<0.01 |
| Sorbitan palmitate | -7.0678 | P<0.01 |
| ST dimethyl60/60/60/30 5Z.7E.22E.24E.24bE-1S.3R-26.27-dimethyl-24a.24b.24c-trihomo-9.10-seco-5.7.1019.22.24.24b-cholestahexaene-1.3.25-triol | -3.8808 | P<0.01 |
| Asp-Leu-Pro-Ser | -4.2175 | P<0.01 |
| Delta 8.14 -Sterol | -4.5842 | P<0.01 |
| PI403 | -3.9259 | P<0.01 |
| 1-O-Caffeoyl-b-D-glucose 6-O-sulfate | -2.3037 | P<0.01 |
| Quinic acid | -2.2392 | P<0.01 |
| 3-heptaprenyl-4-hydroxybenzoate | -5.3764 | P<0.05 |
| Ala-Lys-Thr-Arg | -2.4415 | P<0.05 |
| 3-Hydroxy-2-methylpyridine-4.5-dicarboxylate | -2.6633 | P<0.05 |
| Lysyl-Glycine | -1.4018 | P<0.05 |
| Ergosterol | -5.016 | P<0.05 |
| 629.753811.1642 | -5.1183 | P<0.05 |
| Lys-Trp-Pro | 1.7096 | P<0.05 |
| Canrenone | -2.4051 | P<0.05 |
| N-Undecylbenzenesulfonic acid | -3.2378 | P<0.05 |
| Theonellasterol B | -3.9968 | P<0.05 |

| | | |
|---|---|---|
| Guanosine triphosphate adenosine | 3.4386 | P<0.05 |
| 3-Methylbutyl dodecanoate | -3.7432 | P<0.05 |
| Ala-Asn-Pro-Pro | -4.1391 | P<0.05 |
| 3-Methylthiopropionic acid | -2.1384 | P<0.05 |
| PI426 | -4.843 | P<0.05 |
| N-Acetylhistamine | 1.6269 | P<0.05 |
| Asp-Asp-Cys-Cys | -1.3811 | P<0.05 |
| Arginyl-Hydroxyproline | -1.1013 | P<0.05 |
| S-8-Gingerol | -2.6262 | P<0.05 |
| 17-Beta-Estradiol-3.17-beta-sulfate | -1.0791 | P<0.05 |
| 1-Phospho-alpha-D-galacturonate | 1.3592 | P<0.05 |
| 3-Methylbutyl decanoate | -3.3277 | P<0.05 |
| Berteroin | 1.0577 | P<0.05 |
| Cys-Lys-Gln-Pro | -2.422 | P<0.05 |
| Sinapinic acid-O-sulphate | -1.5291 | P<0.05 |
| Homo-L-arginine | 1.2495 | P<0.05 |
| 2.3-bisAcetyloxypropyl icosanoate | -4.6272 | P<0.05 |
| PR Coenzyme Q8 | -4.6828 | P<0.05 |
| 3-octaprenyl-4-hydroxy-5-methoxybenzoate | -4.9768 | P<0.05 |
| 16-Hydroxy hexadecanoic acid | -1.4103 | P<0.05 |
| Bisnorcholic acid | -1.7036 | P<0.05 |
| 2-Dodecylbenzenesulfonic acid | -2.0799 | P<0.05 |
| PR 5-D-ribonylhopane | -3.5771 | P<0.05 |
| Sulforhodamine B | 2.7691 | P<0.05 |
| Cyclic AMP | -2.4736 | P<0.05 |
